# Lactulose drives a reversible reduction and qualitative modulation of the faecal microbiota diversity in healthy dogs

**DOI:** 10.1038/s41598-019-50090-7

**Published:** 2019-09-16

**Authors:** Marisa da Fonseca Ferreira, Silke Salavati Schmitz, Jeffrey Joseph Schoenebeck, Dylan Neil Clements, Susan Mary Campbell, Donna Elaine Gaylor, Richard J. Mellanby, Adam George Gow, Mazdak Salavati

**Affiliations:** The Royal (Dick) School of Veterinary Studies and The Roslin Institute, The University of Edinburgh, Easter Bush Campus, Roslin, Midlothian EH25 9RG United Kingdom

**Keywords:** Microbial ecology, Liver cirrhosis

## Abstract

Hepatic encephalopathy is a frequent and debilitating complication of liver disorders. Lactulose is an established and reasonably effective treatment, yet with incompletely understood mechanisms of action. The aims of this study were to examine how the faecal microbiota composition changed before, during and after lactulose treatment in a large animal model. Healthy, privately owned dogs (n = 18) completed a prospective cohort study. Faecal samples were collected weekly, while the subjects were either on their usual diet (week 1), or a standardised diet (weeks 2–9), with added oral lactulose in weeks 6–7. DNA extraction and 16S rRNA gene sequencing were undertaken. Faecal samples from week 7 had a significantly lower microbiota richness/diversity, based on observed operational taxonomic units, Shannon/Chao1 indexes and Pielou’s Evenness. Beta diversity based on UniFrac distances was significantly different in week 7 compared to weeks 1, 5 and 9. At the phylum level, week 7 was associated with a significant increase of Firmicutes and Actinobacteria, and a decrease of Bacteroidetes and Fusobacteria, when compared to weeks 5 and 9. In summary, we have shown that lactulose induces a reversible qualitative and quantitative change of the faecal microbiota, which may explain its clinical efficacy in the management of hepatic encephalopathy.

## Introduction

Hepatic encephalopathy (HE) is a frequent and debilitating neurological complication in patients with liver disease. Severe (grades 3–4) HE is associated with higher in-hospital and 30-day mortality, independently of extrahepatic organ failures^1^, and higher liver transplantation 90-day wait list mortality^2^. Covert (minimal and grade 1) HE directly results in human morbidity, being an independent predictor of reduced health-related quality of life and poor sleep quality^3^. Furthermore, HE contributes to a substantial economic burden. In the USA alone, total HE-related hospitalisation charges amounted to $7.245 billion in 2009^4^, and up to $58,625 per patient in 2012^5^.

The pathogenesis of HE is not fully understood^6^. Several neurotoxins and precipitating factors have been implicated, with ammonia being the most well characterised one. In advanced liver disease, this gut-derived neurotoxin may accumulate in the blood and in the brain, due to lack of hepatic conversion into urea and subsequent urinary excretion^7^. Clinically, in human cirrhosis, plasma ammonia has been correlated with both the severity of HE and the frequency of other organ failures, and was identified as an independent predictor of 28-day mortality^8^.

Lactulose, a synthetic non-absorbable disaccharide, is a commonly used medication, with or without the addition of the antibiotic rifaximin, for both the treatment and prevention of HE, with a reasonable evidence of efficacy and added benefits in reducing morbidity and mortality^9,10^. The postulated benefits of lactulose include: (1) decreased colonic transit time and pH, leading to decreased ammonia production and absorption; (2) increased bacterial assimilation of ammonia; (3) decreased bacterial generation of ammonia; (4) production shift from toxic to non-toxic short-chain fatty acids (SCFA); and (5) reduced bacterial DNA translocation^11,12^. Yet, its mechanisms of action remain incompletely elucidated.

Faecal dysbiosis is known to occur in covert and overt HE. Cirrhotic patients with minimal HE (MHE) harbour a higher proportion of urease-producing *Streptococcus salivarius* in stool samples, positively correlating with serum ammonia accumulation^13^. Additionally, the cirrhosis dysbiosis ratio (CDR), a previously validated ratio of autochthonous to non-autochthonous taxa in stool samples of cirrhotic patients, is lowered after development of severe HE, indicating worsened dysbiosis, and associated with 30-day mortality and organ failure^14^. Moreover, the presence of specific bacterial families (*Alcaligenaceae*, *Porphyromonadaceae*, *Enterobacteriaceae*) is strongly associated with poor cognition and inflammation in HE patients^15^. Considering the ongoing evidence regarding microbiome disruption in cirrhosis and HE^16^, it is likely that manipulation of the microbiota may contribute to improved outcomes. Interventions with proposed positive impact have so far included probiotics^17^, diet^18^ and faecal microbiota transplantation^19^.

The impact of lactulose in ameliorating cirrhosis and HE-associated faecal dysbiosis is controversial. A direct impact has been supported by studies based on culture-dependent methodologies, namely: increased *Bifidobacterium*, *Lactobacillus* and *Bacteroidaceae* colonies, and reduced *Enterobacteriaceae*, *Enterococcus* and yeasts in patients with MHE, alongside with improved blood ammonia, psychometric tests and reduced risk of developing overt HE^20^; and increased total aerobic and anaerobic bacterial counts, and lactobacilli in cirrhotic patients without clinical HE, alongside with decreased faecal pH^21^. Conversely, studies based on culture-independent techniques have not substantiated an effect of lactulose in the microbiome of cirrhotic patients without HE^22^ and have reported only a minimal change in cirrhotic patients with HE^14^, including after lactulose withdrawal^23^.

However, no study using next generation sequencing techniques has assessed quantitative and qualitative changes of the intestinal or faecal microbiome, i.e. changes based on both the abundance and the presence or absence of microbial communities, after lactulose treatment in patients entirely naïve to lactulose. The effects of oral lactulose on the faecal microbiome of healthy humans have only been evaluated through either culture-based^24–26^ or culture-independent methods targeting predominant bacterial groups^27–30^. As diet was only standardised in two of those studies^24,30^, it seems likely that a variable diet could have impacted results^31^.

The human faecal microbiome is closer to the canine faecal microbiome when compared to pigs or mice^32^. Dogs evolved to cohabit with people, and hence adapted to a similar diet^33^. As they are frequently kept as companion animals, they are also exposed to the same environment. In addition, dogs can equally suffer from HE and lactulose is frequently used as supportive treatment in this condition^34^. Consequently, companion dogs can represent a useful comparative model to explore the faecal microbiome in HE as well as the effects of certain interventions on its composition, richness and function.

Therefore, the aims of this study were to investigate the magnitude and duration of quantitative and qualitative changes of the faecal microbiota by lactulose in healthy privately-owned dogs fed a standardised commercial diet. It was hypothesised that oral lactulose administration would significantly and transiently change the faecal microbiota in healthy dogs.

## Methods

### Prospective cohort study design

**Figure 1:**
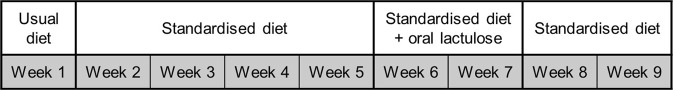
Longitudinal interventions in cohort of healthy owned dogs, designed to assess the faecal microbiota.

Dogs owned by members of staff at the Hospital for Small Animals, the Royal (Dick) School of Veterinary Studies, University of Edinburgh, were recruited with the following inclusion criteria: no current history of any disease; up to date vaccination and deworming records; and no current or recent administration of medications. A faecal sample was requested to be collected from each subject weekly, pertaining to the interventions schematised in Fig. 1.

The standardised diet was a commercial maintenance diet for adult dogs (Hill’s™ Science Plan™ Canine Adult Advanced Fitness™ Large Breed with Chicken, Hill’s Pet Nutrition Ltd., Guildford, UK) and the lactulose was a 3.5 g/5 ml oral solution (Sandoz Ltd, Hampshire, UK). The dose of lactulose was calculated at 0.5 ml/kg and given every 12 hours, unless excessively soft or unformed faeces were noticed, at which point the subjects’ owners would notify one of the authors (MFF) and subsequently decrease the dose by 25% each time, aiming to achieve a soft faecal consistency that would be still amenable for manual collection.

Faecal samples were collected into plain bijoux tubes, kept frozen at −20 °C for a maximum of 24 hours and transferred afterwards to a −80 °C archiving freezer.

Informed consent was obtained from each subject’s owner. The study was approved by the University of Edinburgh’s Veterinary Ethical Review Committee (reference number 112–14) and carried out in accordance with the institution’s relevant guidelines and regulations.

### Faecal DNA extraction, amplification and sequencing

Each sample was defrosted, manually homogenised and DNA extraction performed with a commercial kit (PowerSoil^®^ DNA Isolation Kit, MO BIO Laboratories, Inc., Carlsbad, CA, USA) according to the manufacturer’s instructions^35^. Amplification of DNA was undertaken with PCR of the hypervariable V4 region of the 16S ribosomal RNA (rRNA) gene, using dual-indexing primers (515F/806R), followed by amplicon quantification (Quant-iT™ PicoGreen^®^, Invitrogen, Ltd., Paisley, UK) and pooling, as previously described^36,37^. Standard 16S rRNA library preparation and sequencing with the Illumina^®^ MiSeq^®^ (v2 150PE) platform were performed by Edinburgh Genomics (The University of Edinburgh, UK).

### Data analysis

Software packages for data analysis included the Quantitative Insights Into Microbial Ecology (QIIME2™, https://qiime2.org/) pipeline, RStudio^®^ (version 1.1.453, © 2009–2018 RStudio, Inc., Boston, MA, US) with the package qiime2R (v0.12), as well as GraphPad Prism^®^ (version 7, GraphPad Software Inc., La Jolla, CA, US).

The paired-end raw reads (Supplementary Data Mendeley Data) were analysed using QIIME2™. Briefly, sequence data with sequence quality information was imported through the “fastq manifest” format *PairedEndFastqManifestPhred33*. Demultiplexed sequence counts were summarised showing a median of 58,478 sequences per sample, with a minimum of 27,297 and maximum of 134,220. Sequence quality trimming, chimera filtering and feature table construction was performed through the DADA2 pipeline^38^. Mapping of feature identifiers to sequences was created using links for the Basic Local Alignment Search Tool (BLAST)^39^, and multiple sequence alignment of the sequences was undertaken by the Fast Fourier Transform (MAFFT) program^40^. This was followed by filtering of the alignment with the mask plugin^41^, generation of a phylogenetic tree with the FastTree program^42^ and application of midpoint rooting. The QIIME2™ *q2-diversity* plugin was used for rarefaction analysis and computation of alpha diversity metrics (observed operational taxonomic units [OTUs], Shannon diversity index, Chao1 and Pielou’s Evenness), as well as beta diversity metrics (weighted and unweighted UniFrac distances). Finally, assigning taxonomy to sequences was performed with a pre-trained Naïve Bayes classifier (Greengenes 13_8 99% OTUs) through the QIIME2™ *q2-feature-classifier* plugin^43^.

A human-extrapolated CDR: ratio of commensal autochthonous taxa (*Lachnospiraceae*, *Ruminococcaceae*, *Veillonellaceae*, and Clostridiales Insertae Sedis XIV) to potentially pathogenic non-autochthonous taxa (*Enterobacteriaceae* and *Bacteroidaceae*)^44^, was calculated without the inclusion of Clostridiales Insertae Sedis XIV, as this taxon was not detected in the studied population.

Statistical analyses used included descriptive statistics, as well as the following inferential statistical tests: Kruskal-Wallis test to compare differences between subjects regarding alpha diversity metrics; Wilcoxon matched-pairs signed rank test to compare differences between weeks regarding alpha diversity metrics, taxonomy frequencies and the CDR; paired t-test to compare differences between weeks regarding the presence of *Lachnospiraceae*; and Permutational Multivariate Analysis of Variance (PERMANOVA) test to compare differences between subjects and weeks regarding beta diversity metrics^45^. Normality was assessed with the Shapiro-Wilk test. Statistical significance level was set at *p* < 0.05.

## Results

### Population’s baseline characteristics

A total of 21 dogs were enrolled, with a median age of 5 years (range of 2–10 years). Sex distribution included 12 females (11 neutered) and 9 males (7 neutered). Just over half (n = 11) of the cohort was represented by crossbred dogs, with the remaining 10 dogs distributed as follows: two each of English Cocker Spaniel, English Springer Spaniel, Border Collie and Podenco; and one each of Boston Terrier and Labrador Retriever.

### Adherence to study protocol and side effects related to lactulose administration

Three dogs did not complete the study for the following reasons: acute diarrhoea following dietary indiscretion (n = 1); requirement of a NSAID (meloxicam) for pain management secondary to presumptive degenerative joint disease (n = 1); and lip fold dermatitis after being licked by another dog in the household once starting lactulose (n = 1). In addition, two faecal samples (one each from weeks 4 and 8) were accidentally not collected. In total, 172 faecal samples were analysed, one of which failed sequencing (week 6). Side effects associated with lactulose administration included excessively soft faeces (n = 7) and unformed faeces (n = 1), all resolving after a dose reduction of 25% and 50%, respectively, therefore not requiring a drop out from the study.

### Alpha diversity

**Figure 2:**
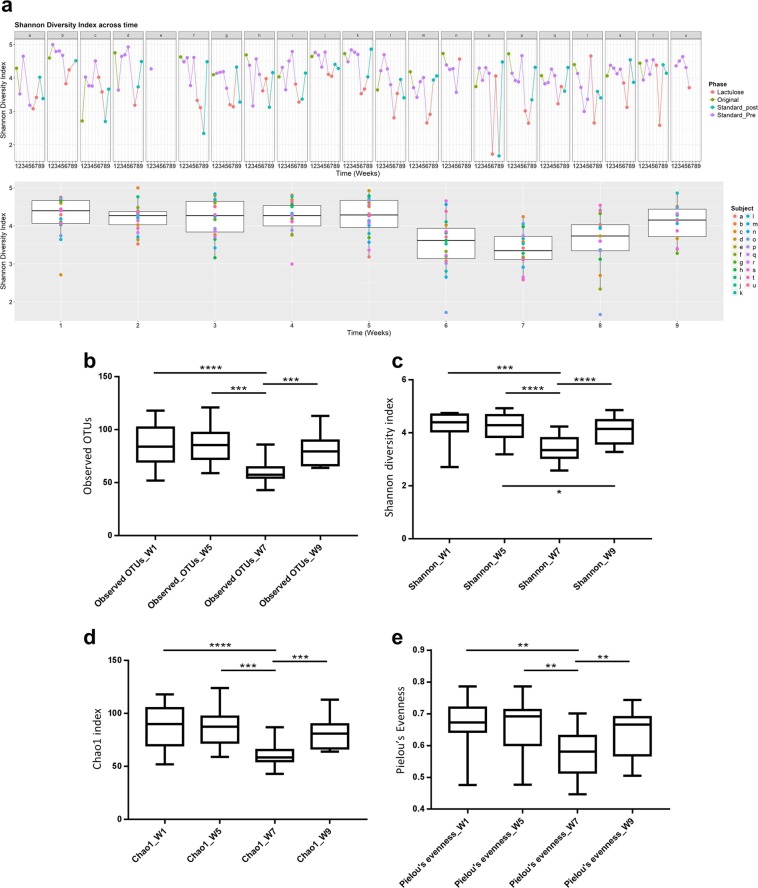
Variation of the canine faecal microbiota, assessed by alpha diversity metrics. (**a**) Shannon diversity index across a cohort of healthy owned dogs (letters a-u) while either on their usual diet (Original, week 1), a standard diet (Standard_Pre, weeks 2–5), a standard diet and oral lactulose (Lactulose, weeks 6–7), or a standard diet after having stopped lactulose (Standard_Post, weeks 8–9). (**b**–**e**) Box and whiskers plots (median, 25^th^ and 75^th^ quartiles), depicting different alpha diversity metrics at selected weeks: observed operating taxonomic units (OTUs) (**b**); Shannon diversity index (**c**); Chao1 index (**d**); and Pielou’s Evenness. (**e**) *p < 0.05; **p < 0.01; ***p < 0.001; ****p < 0.0001 (Wilcoxon matched-pairs signed rank test).

Community richness and diversity values were different between subjects regarding the following metrics: observed OTUs (*p* < 0.0001), Shannon diversity index (*p* = 0.0025), Chao1 index (*p* < 0.0001) and Pielou’s Evenness (*p* < 0.0001). Assessment of community richness and diversity across time is shown in Fig. 2. There was a reduction of all the above metrics at week 7 (standardised diet and oral lactulose), when compared to weeks 1 (usual diet), 5 (standardised diet) and 9 (standardised diet after having stopped lactulose). Conversely, values from weeks 1, 5 and 9 didn’t differ from each other apart from Shannon diversity index values between week 5 and 9 (Table 1).

**Table 1:** Statistical significances (*p*) of alpha diversity metrics for the canine faecal microbiota when compared across time.

Metric	Weeks 1 vs 5	Weeks 1 vs 7	Weeks 1 vs 9	Weeks 5 vs 7	Weeks 5 vs 9	Weeks 7 vs 9
Observed OTUs^a^	0.8056	<0.0001	0.7904	0.0002	0.3516	0.0003
Shannon diversity index	0.9843	0.0003	0.0814	<0.0001	0.0268	<0.0001
Chao1 index	0.7453	<0.0001	0.7379	0.0002	0.2690	0.0003
Pielou’s Evenness	0.7680	0.004	0.0898	0.0010	0.0898	0.009

Cohort of healthy owned dogs while either on their usual diet (week 1), a standard diet (week 5), a standard diet and oral lactulose (week 7), or a standard diet after having stopped lactulose (week 9). Statistical test used: Wilcoxon matched-pairs signed rank test.

^a^Operational Taxonomic Units.

### Beta diversity

**Figure 3:**
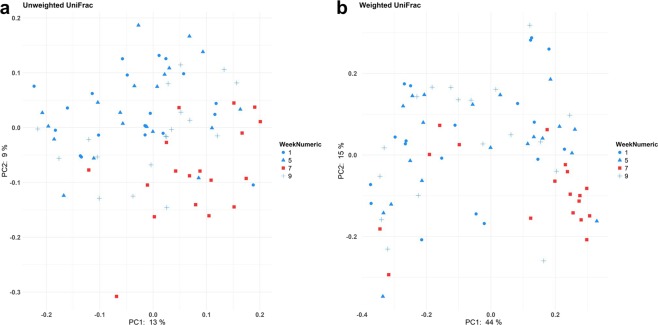
Variation of the canine faecal microbiota, assessed by beta diversity metrics. (**a**,**b**) Principal coordinate analysis based on unweighted (**a**) and weighted (**b**) UniFrac distances. Cohort of healthy owned dogs while either on their usual diet (week 1), a standard diet (week 5), a standard diet and oral lactulose (week 7), or a standard diet after having stopped lactulose (week 9). Samples from week 7 were significantly different (*p* < 0.01, PERMANOVA test) from weeks 1, 5 and 9, while none of the latter differed from each other.

Bacterial community presence/absence (qualitative) and abundance (quantitative) assessments with unweighted and weighted UniFrac distances, respectively, are illustrated in Fig. 3. These were overall different between subjects (*p* < 0.001). When analysed across time, the distances at week 7 were different from weeks 1, 5 and 9. However, values at weeks 1, 5 and 9 didn’t differ from each other (Table 2).

**Table 2:** Statistical significances (*p*) of beta diversity metrics for the canine faecal microbiota when compared across time.

Metric	Weeks 1 vs 5	Weeks 1 vs 7	Weeks 1 vs 9	Weeks 5 vs 7	Weeks 5 vs 9	Weeks 7 vs 9
Unweighted UniFrac	0.534	0.001	0.136	0.001	0.272	0.012
Weighted UniFrac	0.474	0.001	0.978	0.002	0.645	0.001

Cohort of healthy owned dogs while either on their usual diet (week 1), a standard diet (week 5), a standard diet and oral lactulose (week 7), or a standard diet after having stopped lactulose (week 9). Statistical test used: PERMANOVA.

### Taxonomy

**Figure 4:**
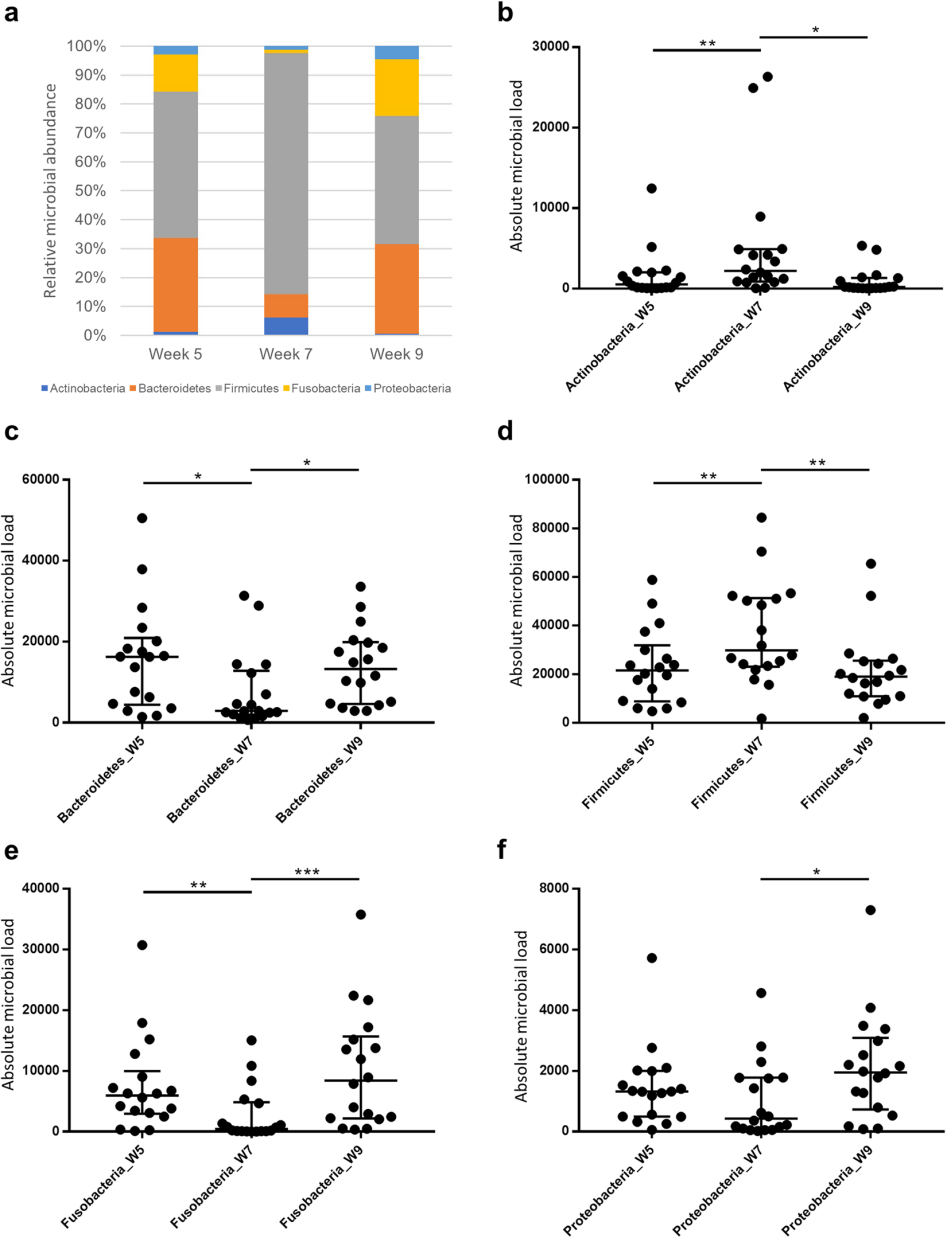
Abundance of phyla in the canine faecal microbiota. Cohort of healthy owned dogs while either on a standard diet (week 5), a standard diet and oral lactulose (week 7), or a standard diet after having stopped lactulose (week 9). (**a**) Median relative abundance of different phyla. (**b**–**f**) Dot plots (including bars for median, 25^th^ and 75^th^ quartiles) depicting absolute abundance of: Actinobacteria (**b**); Bacteroidetes (**c**); Firmicutes (**d**); Fusobacteria (**e**); and Proteobacteria (**f**). *p < 0.05; **p < 0.01; ***p < 0.001 (Wilcoxon matched-pairs signed rank test).

The relative and absolute distribution of phyla abundance across time is depicted in Fig. 4. Irrespective of week number (5, 7 or 9), the most abundant phylum was Firmicutes, followed by Bacteroidetes. A shift of phyla was observed at week 7, with the third most abundant phylum being Actinobacteria, followed by Fusobacteria and Proteobacteria. In contrast, for both weeks 5 and 9, Fusobacteria was the third most abundant phylum, followed by Proteobacteria and Actinobacteria. Moreover, when compared to weeks 5 and 9, week 7 was associated with a higher abundance of both Firmicutes (*p* = 0.0056 and 0.0047, respectively) and Actinobacteria (*p* = 0.0090 and 0.0104, respectively), and lower abundance of both Bacteroidetes (*p* = 0.0304 and 0.0120, respectively) and Fusobacteria (*p* = 0.0040 and 0.0008, respectively). The abundance of these phyla was similar between weeks 5 and 9 (Firmicutes, *p* = 0.6397; Actinobacteria, *p* = 0.6095; Bacteroidetes, *p* = 0.8650; and Fusobacteria, *p* = 0.1964).

**Figure 5:**
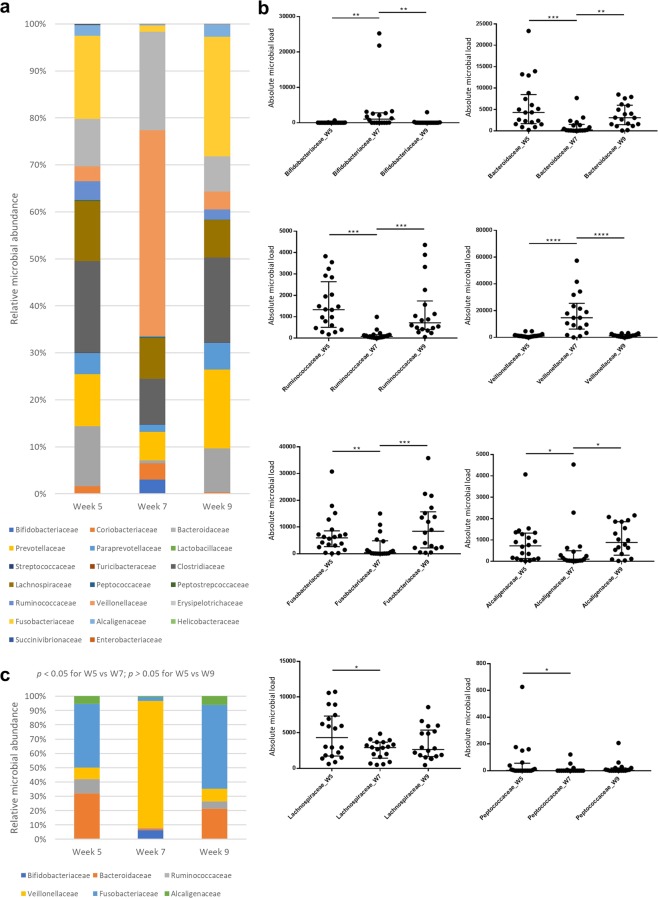
Abundance of families in the canine faecal microbiota. Cohort of healthy owned dogs while either on a standard diet (week 5), a standard diet and oral lactulose (week 7), or a standard diet after having stopped lactulose (week 9). (**a**) Median relative abundance of families with counts present in at least 50% of dogs in at least one week group. (**b**) Dot plots (including bars for median, 25^th^ and 75^th^ quartiles) depicting absolute abundance of statistically significant families (*p < 0.05; **p < 0.01; ***p < 0.001; ****p < 0.0001; Wilcoxon matched-pairs signed rank test). (**c**) Median relative abundance of statistically significant families (Wilcoxon matched-pairs signed rank test).

A total of 20 families were found to be present in the microbiota of at least half of the subjects at one or more time points (Fig. 5a). Different abundances across time were found in eight families (Fig. 5b), six of which showed a different abundance in week 7 when compared to weeks 5 and 9 (Fig. 5c), while both these time points had similar abundances. Significant changes at week 7 included an increased representation of *Veillonellaceae* and *Bifidobacteriaceae*, and a decreased abundance of *Fusobacteriaceae*, *Bacteroidaceae*, *Ruminococcaceae*, *Alcaligenaceae*, *Lachnospiraceae* and *Peptococcaceae*.

### Cirrhosis dysbiosis ratio

**Figure 6:**
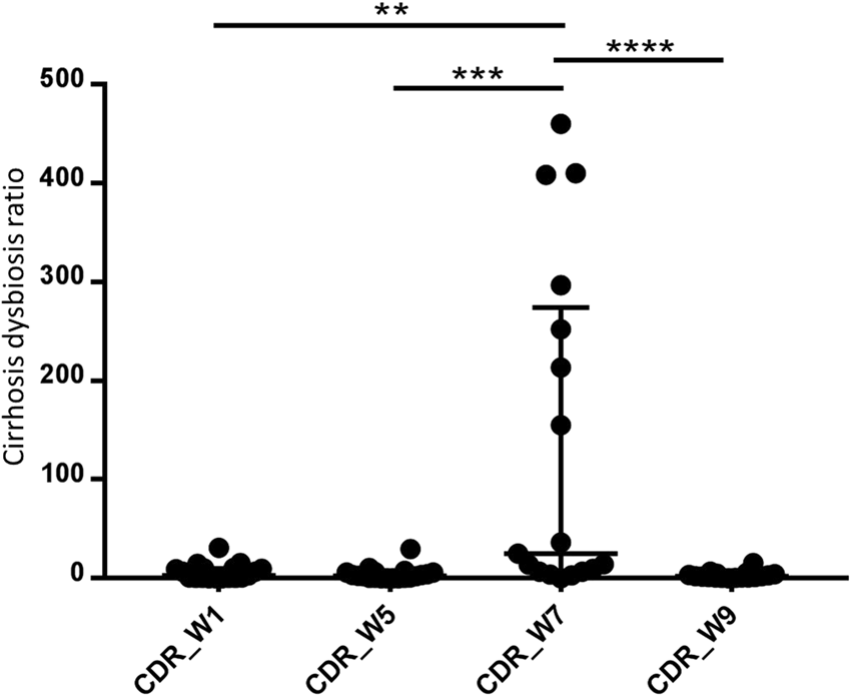
Cirrhosis dysbiosis ratio (CDR), calculated from the canine faecal microbiota, extrapolated from humans. Cohort of healthy owned dogs while either on their usual diet (week 1), a standard diet (week 5), a standard diet and oral lactulose (week 7), or a standard diet after having stopped lactulose (week 9). **p < 0.01; ***p < 0.001; ****p < 0.0001 (Wilcoxon matched-pairs signed rank test).

When analysing the CDR across time (Fig. 6), the highest values were obtained at week 7 with a median of 25.07 (range of 0.08–460.04). These were different from week 1 (median 3.19, range 0.18–30.98, *p* = 0.0079), week 5 (median 2.54, range 0.18–29.78, *p* = 0.0003) and week 9 (median 2.10, range 0.24–15.75, *p* < 0.0001). Conversely, the CDRs calculated for weeks 1, 5 and 9 were similar to each other (*p* = 0.5678 for week 1 vs 5; *p* = 0.2288 for week 1 vs 9; and *p* = 0.5509 for week 5 vs 9).

## Discussion

To date, there are no publications employing untargeted or global culture-independent methodologies to assess the faecal microbiome in healthy people receiving lactulose. In people with HE, further limitations often apply, given the common use of standard of care treatment by the time of study enrolment (lactulose, rifaximin, antacids), precluding an evaluation of the microbiome in non-treated HE^14,18^.

To the authors’ knowledge, this study is the first to investigate the effect of lactulose on the canine microbiota. Collectively, lactulose induced a reversible reduction and qualitative modulation of the faecal microbiota diversity in this population of healthy dogs, while on a commercial standardised diet. Both alpha and beta diversity metrics were affected and specific taxa were implicated in this change.

The impact of lactulose has been studied in healthy mice and pigs through culture-independent methods, most showing an increased alpha diversity, which is in contrast to the present study^46–49^. However, experimental animals are kept in laboratory-controlled conditions and both species are known to have a more distant microbiome from humans relative to dogs^32^. Assessing companion dogs, alongside maintaining the advantage of dietary control, may be therefore valuable given their natural shared environment with humans^31^.

No significant changes of the microbiota were observed in this study due to diet change alone. This is likely explained by the relative similarity of composition of commercial diets (most of the original diets consisted of various brands of dry dog food), in comparison to homemade or raw food^50,51^. There were marked inter-individual differences concerning overall alpha and beta diversity, which was not surprising, given the non-relatedness of the subjects and the diversity of breeds evaluated^52,53^.

Significant changes in the abundance of main phyla were observed with the introduction of lactulose and matched by changes noted at family taxa. Within the Firmicutes and Actinobacteria, *Veillonellaceae* and *Bifidobacteriaceae* increased; and decreases in *Bacteroidaceae* and *Fusobacteriaceae* likely reflect lower abundances of Bacteroidetes and Fusobacteria.

*Veillonellaceae* was the most abundant family after the administration of lactulose. Its significance in liver disease has been controversial, as previous studies have reported both increases and decreases in human cirrhosis with and without HE^13,14^. Bacteria of this family convert lactate to acetate and butyrate^54^. Previously, the latter has been positively correlated with the presence of *Veillonellaceae* in canine faeces^55^. Increases in microorganisms producing these SCFA could be beneficial, as in people, acetate was negatively correlated with pro-inflammatory cytokines in cirrhosis, and butyrate was protective against the development of HE^16^. The administration of lactulose has been associated with increased acetate and butyrate production, together with decreased branch-chained fatty acids isobutyrate and isovalerate^46,47,56^. Similarly to the present study, several others have reported increased numbers of faecal *Bifidobacteriaceae*^24–30^, which produce lactate and acetate^57^, as well as contribute to metabolic cross-feeding, stimulating other butyrate-producing bacteria^58^.

Reductions of both *Fusobacteriaceae* and *Alcaligenaceae* induced by oral lactulose have not been previously reported. This finding is potentially significant, as their presence in the stools of patients with cirrhosis and HE has been associated with worsened inflammation/endotoxaemia and poor performance on cognitive tests^15^. On the contrary, decreases in *Bacteroidaceae*^24,26^ and *Ruminococcaceae*^30^ have been previously reported with lactulose administration. *Bacteroides* spp., are known to produce isovalerate^59^, as well as pro-inflammatory and barrier-disruptive neurotoxins/enterotoxins^60^, and β-glucuronidase^61^, a potential carcinogenic. Reduced faecal activity levels of this enzyme have been demonstrated previously with lactulose administration^26,62^. Besides producing butyrate^63^, *Ruminococcaceae* and *Lachnospiraceae* are also known to produce β-glucuronidase^64^. The lack of a significant increase in *Lactobacillaceae* with lactulose administration has been demonstrated before; hence, this study confirms that it is not a major hallmark of lactulose use^24,25,29,30^.

CDR is a measure of dysbiosis in humans: healthy people are reported to have a higher ratio than patients with cirrhosis, and the presence of HE was associated with an even lower CDR^14^. Although extrapolated from studies in people, the increase of CDR during lactulose administration could represent improvement of dysbiosis, despite an overall lower microbiota diversity and richness.

Limitations of this study include the small number of dogs, the collection of voided faecal samples rather than mucosal or luminal colonic samples, the lack of storage buffer/cryoprotectant and the fact that 16S rRNA gene sequencing allows assessment of taxonomy and abundance, but no species level resolution, nor extrapolation of functional data from the microbiome. However, given the longitudinal study design, each subject could serve as their own control, allowing observation of general trends of microbiota changes. For ethical reasons, invasive collection methods were not employed in these privately owned animals^47,65^. Short-term storage of faecal samples without cryopreservative or buffer is likely to not impact on major phyla distribution^66,67^. To investigate functional changes of the microbiome, high-throughput techniques (whole metagenome shotgun sequencing), metabolomics or metatranscriptomics could have been performed, but this was not within the scope of the current study.

In conclusion, lactulose can drive a reversible quantitative and qualitative modulation of the faecal microbiota in this dog model. Future research is warranted to focus on the investigation of microbiome dynamics in canine models of spontaneous naturally occurring HE (e.g. congenital portosystemic shunts)^68^. This could include similar longitudinal studies to assess effects before and after lactulose treatment or the correlation or comparison with other management strategies. Allowing for more targeted treatment endpoints could not only advance knowledge but also improve outcomes in HE.

## Data Availability

The datasets generated and/or analysed during the current study are available in the Mendeley Data repository, 10.17632/8ctyv86ccp.1.
